# The complete chloroplast genome of *Myricaria prostrata*, a threatened plant in the Qinghai–Tibetan Plateau

**DOI:** 10.1080/23802359.2019.1643803

**Published:** 2019-07-19

**Authors:** Xiaofeng Chi, Faqi Zhang, Shilong Chen

**Affiliations:** a Key Laboratory of Adaptation and Evolution of Plateau Biota, Northwest Institute of Plateau Biology, Chinese Academy of Sciences, Xining, China;; b Qinghai Provincial Key Laboratory of Crop Molecular Breeding, Xining, China

**Keywords:** *Myricaria prostrata*, chloroplast genome, phylogenetic analysis

## Abstract

*Myricaria prostrata* is a critically endangered plant mainly distributed in Qinghai–Tibetan Plateau and adjacent areas. In the current research, we report the complete chloroplast genome sequence of *M. prostrata*. The total length of the genome was 155,230 bp with the GC content of 36.39%. 129 genes including 84 protein-coding genes, 37 tRNA genes, and 8 rRNA genes were annotated. Maximum-likelihood (ML) analysis revealed that *Myricaria* forms a clade with *Tamarix* which showed close relationship with the clade of *Hololachna* and *Reaumuria.*

*Myricaria prostrata* is a prostrate shrub mainly distributed in rocky mountain slopes, sandy places in river valleys, and lakesides of high mountain regions in the Qinghai–Tibetan plateau and adjacent areas with an altitude of 4000–5200 m (Wu et al. 2007). *Myricaria prostrata* is an excellent species for soil and water conservation in plateau due to its characteristics of cold, drought, and salt resistance (Hai et al. 2003). However, the populations of *M. prostrata* is decreasing sharply on account of the snow line elevation and the lake area decreasing on the Qinghai–Tibetan plateau (Wang et al. 2009). Thus *M. prostrata* was listed as a critically endangered plant in the list of wild plants under key protection in Xinjiang Uygur Autonomous Region of China (http://www.iplant.cn/rep/prot/Myricaria%20prostrata). Until now, little research concerning *M. prostrata* was reported. In the current study, we report the completed chloroplast genome of *M. prostrata* based on the next-generation sequencing method. We hope that the chloroplast genome will contribute to molecular phylogeny, genetic improvement, conservation, and sustainable management for this threatened species.

*Myricaria prostrata* was collected from Qinghai, China (35°04′54″N, 93°01′18″E) and the specimen was deposited in the Qinghai–Tibetan Plateau Museum of Biology (Voucher specimen: chen2015179, HNWP). The total DNA was extracted from the fresh leaves through Cetyltriethyl Ammnonium Bromide (CTAB) method (Doyle 1987) with modification. Sequencing was conducted on Illumina HiSeq2500 platform (San Diego, CA, USA), and a total of 24,164,723 paired-end reads were obtained. The chloroplast genome was then assembled by SPAdes (Bankevich et al. 2012), annotated by GeSeq (Tillich et al. 2017). The total length of *M. prostrata* chloroplast genome (MN088847) was 155,230 bp and the GC content was 36.39%. The lengths of two inverted repeats (IR), large single copy region (LSC) and small single copy region (SSC) were 25961, 85026, 18282 bp, respectively. A total of 129 genes were predicted including 84 protein-coding genes, 37 tRNA genes, and 8 rRNA genes. Seventeen genes including seven tRNA (*tRNA-ACG*, *tRNA-CAA*, *tRNA-CAU*, *tRNA-GAC*, *tRNA-GUU*, *tRNA-UGC*, *tRNA-UUC*), four rRNA (*rrn4.5*, *rrn5*, *rrn16*, *rrn23*), six protein-coding genes (*ndhB*, *rpl2*, *rpl23*, *rps7*, *ycf2*, *ycf15*) were duplicated in the IR regions. There were 18 intron-containing genes, one of which (*clpP*) contained two introns.

The completed chloroplast genome sequences of *M. prostrata* together with 13 representative species from Caryophyllales and *Artemisia frigida* (outgroup) were aligned by MAFFT (Kazutaka and Standley 2013) and removed ambiguously aligned sites by Gblocks (Castresana 2000). Maximum-likelihood (ML) analysis was implemented using RAxML-HPC2 on CIPRES Science Gateway (http://www.phylo.org/) based on the GTR + G + I nucleotide substitution model. Phylogenetic analysis showed that *Myricaria* forms a clade with *Tamarix* which is sister to the clade of *Hololachna* and *Reaumuria* (Figure 1).

**Figure 1.**
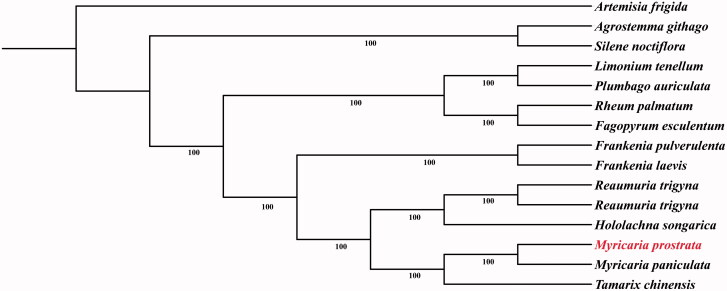
Maximum-likelihood phylogenetic tree based on 15 complete chloroplast genome sequences. Accession numbers: *Agrostemma githago* NC_023357.1, *Artemisia frigida* NC_020607.1, *Drosera rotundifolia* NC_029770.1, *Fagopyrum esculentum* NC_010776.1, *Frankenia laevis* MK397868.1, *Frankenia pulverulenta* MK397869.1, *Hololachna songarica* NC_041273.1, *Limonium tenellum* MK397871.1, *Myricaria paniculata* MK397878.1, *Myricaria prostrata* MN088847, *Plumbago auriculata* NC_041245.1, *Reaumuria trigyna* NC_041265.1, *Reaumuria trigyna* MK397893.1, *Rheum palmatum* NC_027728.1, *Silene noctiflora* NC_016728.1, *Tamarix chinensis* NC_040943.1.
